# Symmetric and antisymmetric forms of the Pauli master equation

**DOI:** 10.1038/srep29942

**Published:** 2016-07-21

**Authors:** A. Y. Klimenko

**Affiliations:** 1 The University of Queensland, SoMME, QLD 4072, Australia

## Abstract

When applied to matter and antimatter states, the Pauli master equation (PME) may have two forms: time-symmetric, which is conventional, and time-antisymmetric, which is suggested in the present work. The symmetric and antisymmetric forms correspond to symmetric and antisymmetric extensions of thermodynamics from matter to antimatter — this is demonstrated by proving the corresponding H-theorem. The two forms are based on the thermodynamic similarity of matter and antimatter and differ only in the directions of thermodynamic time for matter and antimatter (the same in the time-symmetric case and the opposite in the time-antisymmetric case). We demonstrate that, while the symmetric form of PME predicts an equibalance between matter and antimatter, the antisymmetric form of PME favours full conversion of antimatter into matter. At this stage, it is impossible to make an experimentally justified choice in favour of the symmetric or antisymmetric versions of thermodynamics since we have no experience of thermodynamic properties of macroscopic objects made of antimatter, but experiments of this kind may become possible in the future.

Microscopic objects are governed by the equations of quantum mechanics and involve both particles and antiparticles. These equations are time-reversible and do not discriminate between the past and the future^1^,^2^. Nonetheless, the macroscopic objects, which are common in our day-to-day lives, are subject to the laws of thermodynamics that are irreversible in time. The second law of thermodynamics predicts irreversible increase in entropy and, thus, strongly and unambiguously discriminates the directions of time^2^,^3^,^4^: the direction of the thermodynamic time points to the direction of entropy increase. There is another apparent asymmetry in the Universe: macroscopic objects are exclusively made of matter (particles). Antimatter, which is formed by antiparticles in the same way as matter is formed by particles, is theoretically possible but seems not to be present anywhere in the known Universe^5^. While the microscopic properties of antiparticles are generally well known, the fundamental conceptual problem that we are facing is the extension of macroscopic properties from matter to antimatter. We expect that properties of matter and antimatter are in some way similar, but it appears that extension of the second law of thermodynamics from matter to antimatter is not unique, allowing for two possible alternatives: time-symmetric and time-antisymmetric^6^. The thermodynamic times of matter and antimatter run in the same direction according to the former, and in the opposite directions according to the latter. These two possibilities are referred to in the paper as the symmetric and antisymmetric versions of thermodynamics. These versions are mutually incompatible and, since we do not have any experience with macroscopic antimatter, it is not known which one of these versions is real.

One can expect that a quantum (or classical) system of a sufficiently large dimension and complexity should display thermodynamic properties. The apparent discrepancy between the irreversible equations of thermodynamics and reversible equations of classical and quantum mechanics are mitigated by so-called kinetic (or master) equations^2^. Equations of this type are derived from the time-reversible equations of classical or quantum mechanics, but necessarily involve assumptions discriminating the direction of time. If entropy evolved by master/kinetic equations increases forward in time, then these equations are consistent with thermodynamics. The important statements demonstrating monotonic increase of entropy are traditionally called H-theorems after the famous theorem by Ludwig Boltzmann^7^, who demonstrated increase of entropy in gases under the conditions of validity of the hypothesis of molecular chaos (the *Stosszahlansatz*). This hypothesis discriminates the direction of time by assuming that the parameters of molecules are uncorrelated before (but not after) their collisions. The gap between unitary (time-reversible) quantum mechanics and thermodynamics was bridged by Wolfgang Pauli^8^, who derived a master equation by assuming decoherence of the states of a large quantum system before (but not after) unitary interactions of the states takes place. This class of equations, which is referred to as the Pauli master equations (PME), is consistent with thermodynamics: it tends to increase entropy and leads to microcanonical distributions. Demonstration of validity of the corresponding H-theorem was one of the main goals of Pauli’s article^8^.

While there have been many attempts to improve or generalise PME^2^, it seems that some of the more recent changes brought into our understanding of PME have had more of a philosophical or methodological character. While in the early days of quantum mechanics randomisation of quantum phases was viewed as an additional assumption contaminating the scientific rigor of quantum equations^9^, a more modern treatment of this problem^2^,^10^ is that decoherence is a real physical process, whose exact mechanisms are not fully known at this stage. This process primes the direction of thermodynamic time and causes entropy increase. Joos^10^ noted that one of Pauli’s^8^ remarks can be interpreted as pointing to environmental interference. Smaller quantum systems are subject to decoherence due to interference of the environment, while very rapid decoherence of larger systems (i.e. macroscopic objects) is often seen as being indicative of the presence of intrinsic mechanisms of decoherence^11^. While it seems that both mechanisms of decoherence (i.e. environmental and intrinsic) are possible and both are discussed in the literature^11^, we are interested in the consequences of decoherence and do not dwell on its physical causes.

While the symmetric version of thermodynamics is conventionally implied in publications, the possibility of the antisymmetric version raises a number of questions. First, there must be a corresponding antisymmetric version of the PME, which is to be derived from the conventional unitary equations of quantum mechanics. This derivation endeavours to examine the link between macroscopic and microscopic symmetries. Consistency of different versions of PME with the corresponding versions of thermodynamics demands validity of the relevant H-theorems. The question of properties of the derived equations, especially whether PME statistically favours conversion of matter into antimatter or antimatter into matter or is neutral with respect to this conversion, is of particular interest.

## A generic quantum system and perturbation analysis of its unitary evolution

### System Hamiltonian

While considering evolution of a generic quantum system, it is common to distinguish two components in its Hamiltonian:


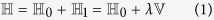


the leading time-independent component 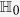 determining the energy eigenstates


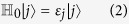


and the disturbance 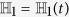 that characterises interactions of these eigenstates |*j*〉 and may be time-dependent^2^,^8^,^9^,^12^. Different states (i.e. eigenstates) may belong to the same energy levels 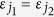, but these states are still marked by different indices *j*_1_ ≠ *j*_2_. The characteristic time associated with the leading Hamiltonian is much smaller than that of the interactions: 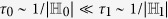 and a small parameter, 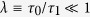, is used in (1) to explicitly reflect that 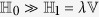. Here, 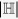 denotes a norm estimate for the Hamiltonian 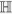. The energy eigenstates form a complete orthogonal basis 〈 *j*|*k*〉 = *δ*_*kj*_ where *ε*_*j*_ represents energy of the *j*^th^ eigenstate. The number of eigenstates, 2*n*, is presumed large (eigenstates can be continuous). The Hamiltonian disturbance 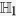 is responsible for interaction of the eigenstates due to non-diagonal elements. If 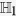 depends on time, the characteristic time of this dependence is assumed to be of order of *τ*_1_. The diagonal elements of 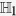 are not of interest and we put


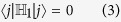


Mathematically, this assumption simplifies the analysis but does not restrict generality since the diagonal elements of 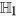 can always be merged with the diagonal elements of 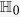.

### Invariant properties of the system

Two types of states are distinguished: the matter states and the antimatter states. The matter states are indexed by 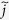, 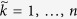, while the antimatter states are indexed by 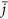, 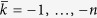. The indices *j* and *k* run over all 2*n* states. We also use the indicator function *C*_*j*_ = ±1 defined by


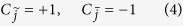


If 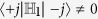, the Hamiltonian (1) allows for conversion between the corresponding matter states and antimatter states. The states |+*j*〉 and |−*j*〉 are presumed to represent the CP transformations of each other


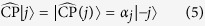


although the physical coordinates and their parity (P) transformation, which reverses the directions of these coordinates, are not explicitly considered here. The CP transformation also involves the charge conjugation (C), which changes particles into antiparticles and wise versa. The time reversal operator (T) reverses the direction of time. The complex phase |*α*_*j*_| = 1 is subject to a number of physical constraints and, in most cases, can be eliminated by incorporating the phase angle into the states. Since our consideration is generic, we keep the phases *α*_*j*_, which, however, can be omitted as they do not affect our consideration and results.

Under conditions considered here, the CP- and CPT-invariant Hamiltonians satisfy:


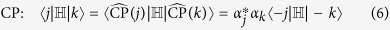



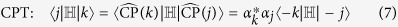


These symmetric properties of quantum systems are discussed in standard monographs^13^,^14^.

It must be noted that not all matter and antimatter states can evolve into each other (or form a quantum superposition), since evolution of quantum systems must preserve a number of conservative properties (such as the electric charge). Hence only mutually convertible states, which preserve the conservative properties, are considered here. Mutual conversions of matter and antimatter inevitably alter the baryon numbers (for example, conversion of a neutron into an antineutron changes this number from +1 to −1). The possibility of the baryon number violations is known as the first Sakharov^15^ condition of baryogenesis and is conventionally presumed in theories dealing with the balance of matter and antimatter.

### Asymptotic expressions for unitary evolution

The evolution of the quantum system is governed by the Schrodinger equation


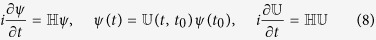


where *ψ*(*t*) is the wave function, the Hamiltonian 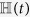 is Hermitian, the evolution operator 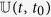 is unitary so that its time inverse corresponds to its conjugate transpose 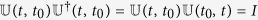, where *I* is the unit matrix.

In quantum perturbation theory^8^,^9^,^12^,^16^, the time evolution operator is conventionally represented by the series 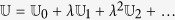 that after substitution into equation (8) yields


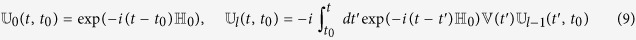


While the interaction term 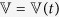 may depend on time, it is assumed that the characteristic time *τ*_*v*_ associated with this change is 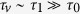. The terms in the expansion can be easily evaluated when Δ*t* = *t* − *t*_0_ is sufficiently small; specifically, assuming that 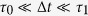 is sufficient for the derivation of PME. In this case we write 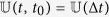 implying that there also exists a weak dependence of 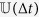 on *t*. Evaluation of the non-diagonal elements results in


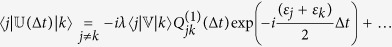



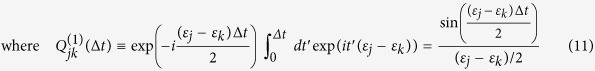


The diagonal elements take the form


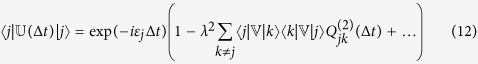



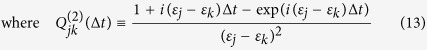


Since 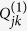 and 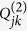 are linked by the equation


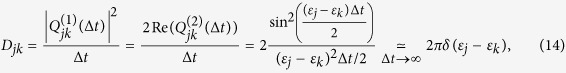


where new quantities *D*_*jk*_ = *D*_*kj*_ forming a symmetric matrix are introduced for convenience. The derived approximations are consistent with the unitary property


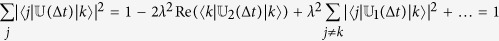


By default, the sums over indices *k* and *j* are evaluated over all 2*n* eigenstates. It is easy to see that, at the leading orders (up to *O*(*λ*^2^)), the magnitudes 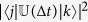 form a symmetric matrix since 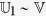 is Hermitian. This property, however, is not valid at the higher orders: generally, the matrix 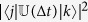 is not symmetric.

While the asymptotic representations of 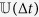 given above are universal, these representations do not define uniquely the form of the master equation, which depends on additional assumptions. Large systems are subject to the process of decoherence, whose properties determine probabilistic behaviour of the system.

## Different forms of the Pauli master equation

This section derives two alternative forms of Pauli master equation (PME): conventional symmetric and non-conventional antisymmetric. In the next section these forms will be shown to correspond to the symmetric and antisymmetric extensions of thermodynamics. PME was originally suggested by Pauli^8^, and has been repeatedly re-derived using varying techniques and assumptions^2^,^9^,^12^,^17^. The original approach developed by Pauli is most suitable for the derivations in this section for a number of reasons. First, Pauli’s approach explicitly discriminates the directions of time by repeated application of decoherence, which corresponds to setting the state of random phases at the beginning of many sequential time intervals. Since human intuition is deeply linked to inequality of directions of time it is common to introduce discrimination of directions of time implicitly by implying “good” initial conditions. As remarked by Price^4^, this implicit treatment is not desirable in applications, where the direction of time needs to be analysed and not postulated a priori. Second, Pauli approach is based on wave functions, which seem to be more convenient for the present analysis, which involves multi-time correlations, than density matrices.

### Symmetric PME

**Figure 1:**
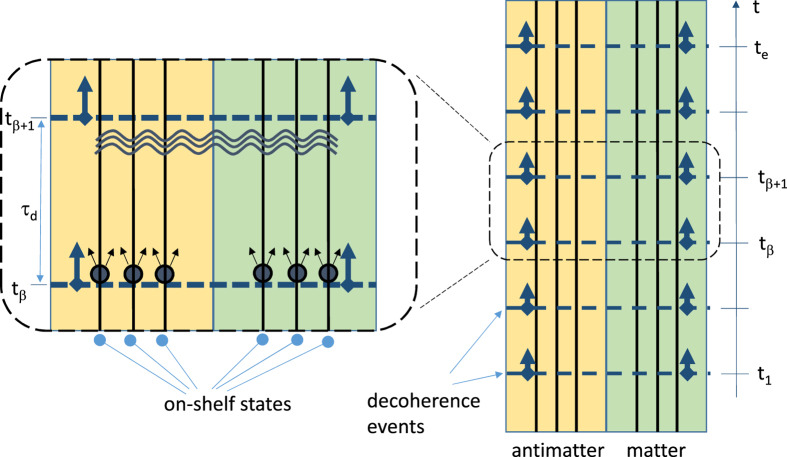
Evolution of a quantum system with uni-directional (symmetric) decoherence events separating intervals of unitary evolution. The vertical lines show states with the same energy. The circles indicate random phases after decoherence. Multiple waved lines indicated a mixture of wave functions.

According to Pauli’s approach to the master equation, the decoherence events occur at the times *t*_0_, *t*_1_, …, *t*_*β*_, …, *t*_*e*_, which, as illustrated in Fig. 1, are spaced by the characteristic decoherence time (*t*_*β*+1_ − *t*_*β*_) ~ *τ*_*d*_. The characteristic decoherence time *τ*_*d*_ is presumed to satisfy


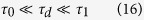


for reasons discussed below. In the symmetric case decoherence occurs forward in time for all (matter and antimatter) states. The energy eigenstates form the preferred basis for decoherence: the phase of a decohered eigenstate becomes independent of the rest of the distribution. The effect of the decoherence on the density matrix *ρ*(*t*) is removing all non-diagonal elements (as specified by the Zwanzig projection operator^18^):


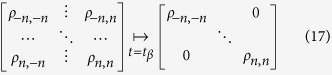


Irrespective of the previous state of the system, this corresponds to transformation of the wave function into a mixture


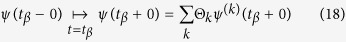


where 2*n* random phases Θ_*k*_ indicate that *ψ* at *t* = *t*_*β*_ + 0 is not a superposition but a mixture of 2*n* wave functions *ψ*^(*k*)^. Each function *ψ*^(*k*)^ in (18) corresponds at *t* = *t*_*β*_ + 0 to the *k*^th^ diagonal term of the density matrix *ρ*_*k*,*k*_ = 〈*ψ*^(*k*)^|*ψ*^(*k*)^〉 and satisfies


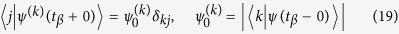


Here, we use random phases Θ_*k*_ as notation that indicates mixed states of quantum system^19^. In this case, Θ_*k*_ can be interpreted as special quantum states. This is not exactly the same but very close in its measured effect to Pauli’s work where phases were assumed to be physically randomised. At the moment *t* = *t*_*β*_ decoherence converts the overall wave function *ψ* of the system (which can be in any state, mixed or pure, at *t* < *t*_*β*_) into a mixture of 2*n* pure states corresponding to the eigenstates of 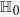. The decoherence events change phases but not the amplitudes of the wave functions. If 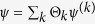, then the magnitude of *ψ* is given by |*ψ*| = 〈*ψ*|*ψ*〉^1/2^, where 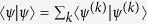.

Due to linearity of the quantum evolutions governed by (8), each function *ψ*^(*k*)^(*t*) evolves independently within each time interval *t*_*β*_ < *t* < *t*_*β*+1_. Specifying Δ*t* = *t*_*β*+1_ − *t*_*β*_ allows us to determine


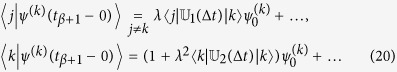


We introduce probabilities


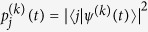


and kinetic coefficients


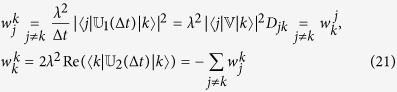


where *D*_*jk*_ is evaluated in (14) and Hermitian properties of 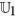 and 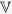 are taken into account to establish that 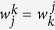 in (21). Here, we assume that 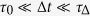, where *τ*_Δ_ ~ 1/Δ*ε* is proportional to the characteristic density of quantum levels in the energy space (the characteristic energy distance Δ*ε* between quantum levels is very small in large systems). The probability change for every *p*^(*k*)^ over the interval Δ*t* = *t*_*β*+1_ − *t*_*β*_ is determined by equations (19, 20, 21) and takes the form


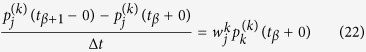


With introduction of the overall probability,


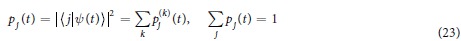


and taking into account that Δ*t* is small, equation (22) is summed over all *k* to give the Pauli master equation^8^


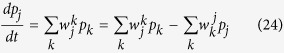


The the right-hand side form of the equation explicitly involves formula for 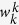 in (21).

### Antisymmetric PME

**Figure 2:**
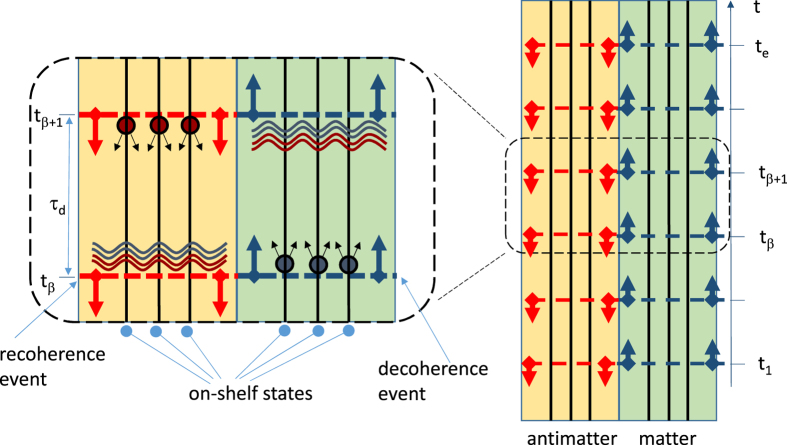
Unitary evolution of a quantum system with counter-directional (antisymmetric) decoherence events (foward in time for matter states on the right-hand side and backward in time for antimatter states on the left-hand side). Notations are similar to Fig. 1.

In the case of antisymmertic decoherence, the matter states decohere at the moments *t*_0_ < *t*_1_ < … < *t*_*β*_ < … < *t*_*e*_ but the antimatter states recohere at the same moments (recoherence is decoherence backwards in time *t* — this is illustrated in Fig. 2. Within every unitary evolution interval *t*_*β*_ ≤ *t* ≤ *t*_*β*+1_, the joint effect of matter decoherence at *t* = *t*_*β*_ and antimatter recoherence at *t* = *t*_*β*+1_ is representation of the wave function in the following form


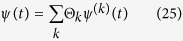


where 2*n* random phases Θ_*k*_ of the decohered values are statistically independent from each other, while the corresponding 2*n* wave functions *ψ*^(*k*)^(*t*) are subject to unitary evolution within the interval interval *t*_*β*_ ≤ *t* ≤ *t*_*β*+1_ and satisfy the boundary conditions


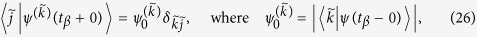



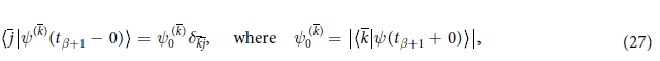



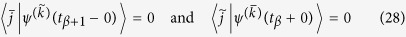


Note that the property specified by these equations cannot be expressed in terms of the conventional single-time density matrix *ρ*(*t*), since correlations at different time moments are needed in (25)–(28). In general, this problem requires consideration of a two-time density matrix (e.g. *ρ*(*t*_1_, *t*_2_) = |*ψ*(*t*_1_)〉 〈*ψ*(*t*_2_)|) but using wave functions seems to be more convenient and is perfectly sufficient for our goals. Note that, unlike in the case of symmetric decoherence, the antimatter states have coherent components at *t* = *t*_*β*_ + 0 just as the matter states have coherent components at *t* = *t*_*β*+1_ − 0.

While the formulae (8)–(14) for the unitary evolution operator 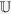 are the same as in the symmetric case, the wave function *ψ*(*t*), which is evaluated below, is different from (20) due to differences in the boundary conditions. While some of the terms remain similar to (20) at the leading order


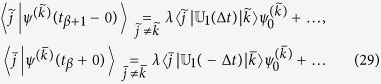


the other terms change


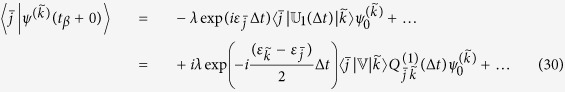



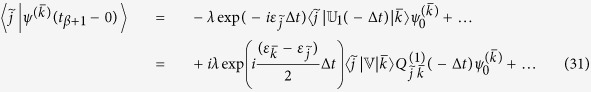


to ensure compliance with the respective boundary conditions in (28). The additional exponential multipliers exp(±*iε*_*j*_Δ*t*) appear due to the phase change between the states | *j*〉 taken at *t* = *t*_*β*_ + 0 and *t* = *t*_*β*+1_ − 0. As previously, the real parts of the diagonal terms must be evaluated up to the second order


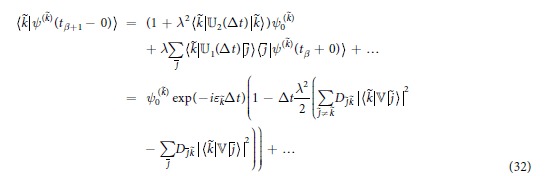


The diagonal contributions from the antimatter states are evaluated in a similar manner


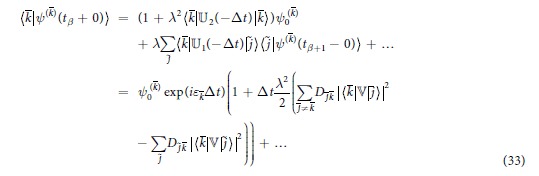


Taking squares of the the wave functions results in


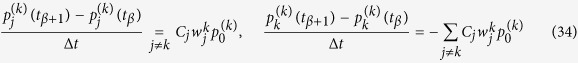



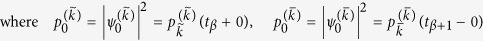


and the coefficients 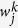 are still specified by (21). Evaluation of the sum 
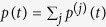
, while taking into account that *p*(*t*_*β*_ + 0) = *p*(*t*_*β*_ − 0) is continuous (for any *t*_*β*_) and that 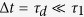 is small, yields the master equation


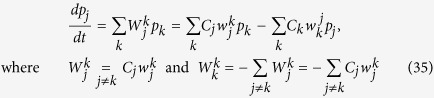


As previously the coefficients of this master equation can depend on time 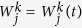. The off-diagonal elements differ from those in SPME only by their signs 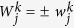 for *j* ≠ *k* but the diagonal elements are generally different 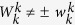. In absence of matter the evolution of the antimatter states according to APME represents, as expected, a time reversal of the evolution of these states according to SPME. Note that, in addition to this expected result, the derived equation also evaluates another, highly non-trivial statistical property — how the matter and antimatter states interact with each other.

## Comparison of the two forms of PME

First we note that the forms of PME, symmetric (24) and antisymmetric (35), can both be written as


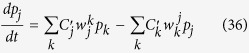


where the modified indicator-function 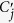 is defined differently for symmetric PME (SPME) and antisymmetric PME (APME) by


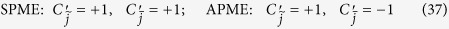


This form is useful for analysis of the common features of these equations.

### Invariant properties

These properties can be summarised by the following proposition:

**Proposition 1** *If the system Hamiltonian is invariant* (*i*.*e*. *CP-invariant or CPT-invariant or both*), *the symmetric Pauli master equation* (*SPME*) *is CP-invariant and the antisymmetric Pauli master equation* (*APME*) *is CPT-invariant*.

First we note that both constraints imposed on the Hamiltonian, (6) and (7) are the same for diagonal elements *j* = *k* resulting in *ε*_*j*_ = *ε*_−*j*_. For off-diagonal elements, the CP invariance yields 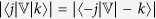 so that 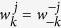, while the CPT invariance yields 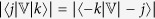 so that 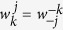. Here we use the definition of 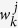 by (21) and take into account that |*α*_*j*_| = 1 in (6) and (7). Finally, the symmetry of the kinetic coefficients 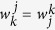 determines that both of the invariant properties, CP in (6) and CPT in (7), result in the same set of constrains


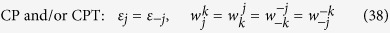


While in general the constraints imposed on the interaction Hamiltonians by CP invariance and by CPT invariance produce different evolutions of quantum systems, this difference is not revealed in the PME when the decoherence time is sufficiently short, as stipulated by (16). The statement of the proposition is then easily proved by substituting −*j* for *j* in (24) and −*j* for *j* and −*t* for *t* in (35). This proposition indicates that SPME can be referred to as the CP-invariant PME and APME can be referred to as the CPT-invariant PME, although using these terms should not lead to confusion of invariant properties of PME with those of the Hamiltonian (and with symmetries of unitary evolutions corresponding to the Hamiltonian). The same statement applies to the two possible extensions of thermodynamics.

### Consistency with thermodynamics

The definition of entropy


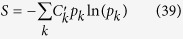


coincides with the conventional definition of entropy for SPME but changes the signs of contributions of the antimatter states for APME. This definition of entropy does not involve the degeneracy factors, as the summation is performed over all quantum states (and not over different energy levels, which can be degenerate). Note that the placement of 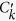 in (36) and (39) does not allow for interpretation of 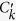 as effective degeneracy factors. The entropy *S* defined by (39) satisfies the following H-theorem:

**Proposition 2** *The entropy S monotonically increases in evolution of probabilities predicted by the Pauli master equation* (*both symmetric and antisymmetric*), *unless the system is in equilibrium where the entropy remains constant*.

The proof of the proposition is achieved by evaluating *dS*/*dt* using (36)


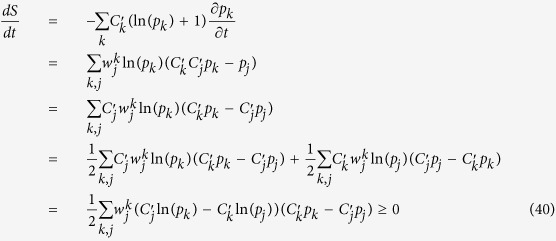


Here we use the normalisation of *p*_*j*_ in (23), equivalence of the summation indices *j* and *k* as well as the symmetry of the coefficients 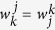 defined in (21). We also note that 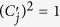 and 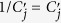. Each term (*j*, *k*) in the last sum is no less than zero — this can be easily seen by considering two alternatives 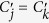 and 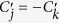. The symmetric version of this H-theorem (i.e. all 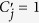), which is conventional^8^, also requires that the kinetic coefficients are symmetric 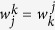. The proof given here is suitable for both SPME and APME.

The essential property of PME is consistency with thermodynamics as stipulated in the following proposition.

**Proposition 3** *Pauli master equations* (*PME*) *are consistent with thermodynamics*: *symmetric PME corresponds to symmetric extension of thermodynamics from matter to antimatter and antisymmetric PME corresponds to the antisymmetric extension of thermodynamics from matter to antimatter*. *Specifically*, *this consistency implies*:

1. *Preserving the overall probability* 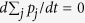;

2. *Preserving the overall energy dE*/*dt* = 0, 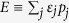;

3. *Entropy definitions that are consistent with the corresponding versions of thermodynamics and obey dS*/*dt* ≥ 0;

4. *Symmetry of the kinetic coefficients* 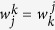 *and* 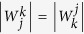.

The first property directly follows from PME (36). The second property is valid since 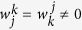 only if *ε*_*k*_ = *ε*_*j*_ in (21), which is valid when decoherence does not interfere with the main eigenstates 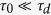. The states with the same energy as the initial state are conventionally called “on-shelf states”. If *τ*_*d*_ is too small, equation (14) can allow for transitions between states with different energies *ε*_*k*_ ≠ *ε*_*j*_ since *D*_*jk*_ in (21) deviates from 2*πδ*(*ε*_*j*_ − *ε*_*k*_) or, alternatively, can freeze any evolution of the system since the change in probabilities is proportional to Δ*t*^2^ when *τ*_*d*_ is very small (these are quantum anti-Zeno and Zeno effects – see ref. 17). The third property: the H-theorem is proven above while the definition of entropy in (39) can be rewritten as


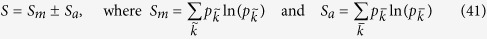


and the plus sign in (41) corresponds to SPME and is consistent with the conventional definition of entropy *S* = *S*_*m*_ + *S*_*a*_ in the symmetric version of thermodynamics. The minus sign corresponds to APME and matches the definition of apparent entropy in the antisymmetric version of thermodynamics^6^, where intrinsic entropies of matter *S*_*m*_ and antimatter *S*_*a*_ are summed up with the opposite signs *S* = *S*_*m*_ − *S*_*a*_.

The symmetry of the kinetic coefficients 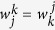 reflects the principle of detailed balance and follows from (21), which is valid when the decoherence time is sufficiently short 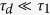 (note that the matrix 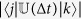 is generally not symmetric when Δ*t* is large). The condition 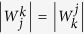 follows from 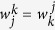 and (35). The equilibrium distribution achieved by SPME corresponds to equal probabilities of all interacting states (i.e. to the microcanonical distribution). As the size of a quantum system increases, the decoherence time is expected to decrease, becoming very small for macroscopic objects — this makes the system behaviour consistent with thermodynamics. Note that the kinetic coefficients 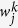 do not depend on the decoherence time *τ*_*d*_ as long as *τ*_*d*_ stays within its expected physical range 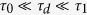. Since not much is known about the exact values of decoherence time, independence of *τ*_*d*_ adds robustness to PME. However, an increase of decoherence time in (16) towards *τ*_*d*_ ~ *τ*_1_ would lead to the need of evaluating higher order terms in the expansion for 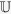, compromising the symmetry of the kinetic coefficients. This would represent a thermodynamic violation, at least, due to violating detailed balance in the microcanonical distributions.

### The beginning of time and the end of time

A solution of SPME can be extended forward in time and backward in time. While this can be done forward in time without encountering any problems, the backward extension has to be terminated as soon as the probability of one of the interacting states becomes zero, otherwise the probabilities predicted by SPME become negative, which is unphysical. This event, when the solution cannot be extended further into the past, is called the beginning of time. Physically, the beginning of time means that either the system is subject to external influence (such as setting the initial conditions) that makes the governing equations invalid or interactions of the states are terminated 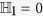 and probabilities become frozen in time. If the direction of thermodynamic time is reversed, the system should experience the end of time instead of the beginning of time events. Since APME runs thermodynamic time in opposite directions for matter and antimatter, it is clear that APME can experience both types of events, the beginning of time and the end of time. Physically these events mean terminations of the interactions between states and/or external interferences.

### Interactions between the matter states and antimatter states

While the interactions within antimatter states can be easily determined due to the reversed-time similarity with interactions of the matter states, it is the interaction between the matter and antimatter states that is the most interesting and non-trivial question to be answered by PME. These interactions can be illustrated by a simple system that has only two quantum energy eigenstates: the matter state *j* = +1 and the antimatter state *j* = −1. The PME for this system take the forms


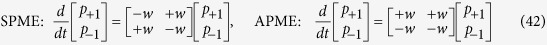


where *p*_+1_ + *p*_−1_ = 1 and evolution is determined by a single kinetic coefficient *w* = *w*(*t*). We assume that *w* > 0, i.e. the superselection rules allow for conversion of matter into antimatter and back. If 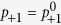 and 
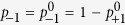
 at the initial moment *t* = *t*_0_, the solutions of equation (42) are given by


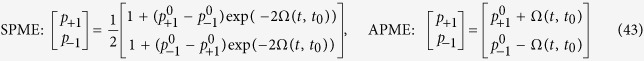



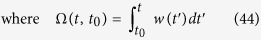


In many cases, Ω(∞, −∞) is quite small for a single interaction event. As expected, when Ω becomes sufficiently large, the SPME solution converges to the state with maximal entropy *p*_+1_ = *p*_−1_ = 1/2. That is, if applied on a large scale, this model predicts equilibration of matter and antimatter that, at equilibrium, should be present in equal proportions. The APME prediction is radically different — this equation transfers probability from the antimatter states to the matter states until the antimatter states can no longer be present (have zero probability). On a large scale, this model predicts that there could not be equilibrium balance between matter and antimatter unless antimatter is fully converted into matter. This can be summarised in form of the proposition:

**Proposition 4** *Assuming that transitions between matter and antimatter states are possible*, *the symmetric form of the Pauli master equation* (*SPME*) *predicts evolution towards equilibrium with the same probability of matter and antimatter*, *while the antisymmetric form of the Pauli master equation* (*APME*) *predicts evolution towards complete conversion of antimatter into matter*.

The evolution specified in the proposition follows from the H-theorem and the definition of entropy by equation (39). The maximal value of entropy (subject to physical constraints) is achieved 1) for SPME when *p*_*j*_ > 0 are the same for all interacting on-shelf states and 2) for APME when *p*_*j*_ > 0 are the same for all interacting on-shelf matter states and *p*_*j*_ = 0 for all antimatter and remaining matter states. The statement of the proposition does not contradict the declared similarity of matter and antimatter. In fact, both SPME and APME are based on similarity of matter and antimatter but interpret this similarity differently. In case of APME (but not SPME) this interpretation involves a time reversal: antimatter is converted into matter forward in time but, in reversed time, the same process converts matter into antimatter.

## Discussion

While conventional flow of time is deeply imbedded into our intuition, it was Boltzmann^7^ who connected the perceived direction of the “flow of time” with the second law of thermodynamics. He suggested that if there was a section of Universe where entropy decreases in time, the local population would perceive the past as the future and the future as the past in that part of the Universe. Even now this statement would seem very strange to many people. APME appeals to similar ideas and, for many people, may contradict the intuitive perception of time, which makes the use of this model more difficult. This is a disadvantage, but there are advantages associated with APME and the antisymmetric (or CPT-invariant) approach to the thermodynamics of antimatter. First, it connects two fundamental asymmetries of our world—the absence of antimatter and the preferred direction of thermodynamic time—by predicting that conversion of antimatter into matter forward in our time is strongly favoured by thermodynamics. Demonstrating that APME tends to convert antimatter into matter is one of the major results of the present work (assuming that matter/antimatter conversions are allowed by the superselection rules — see the previous section).

The microscopic world is mostly CP-invariant. This world inevitably interacts with thermodynamic surroundings and these interactions should also be CP-symmetric. We see macroscopic effects of these interactions in form of thermodynamic irreversibility but do not detect microscopic effects of these interactions, since they do not conflict with the quantum CP invariance. However environmental interactions that generate thermodynamic time can become visible at a microscopic level in CP-violating systems. These interactions are seen as apparent CPT violations even if the quantum system is strictly CPT-preserving^19^. Under these conditions, the ubiquitous nature of thermodynamic interactions may lead to questioning CPT invariance. The second advantage is that the antisymmetric approach offers a very convenient interpretation that strictly upholds the CPT invariance: the apparent CPT violation appears only because exact application of the CPT transformation requires to change environmental matter into antimatter, which is practically impossible.

The system considered in the present work is subject to much stronger thermodynamic interference than that considered in the CP-violating Kaon decays^19^. This strong and persistent influence of decoherence in the derivation of PME ensures thermodynamic compliance for the evolution of the system but suppresses the difference between CP- and CPT-invariant Hamiltonians. It seems, however, that invariant properties of decoherence, which remain largely unknown, should have some physical links with invariant properties of the microscopic world. The effect of decoherence on simulations of realistic interactions of particles and antiparticles should involve radiation and may need to incorporate relativistic quantum mechanics, where differences between microscopic symmetries can be more persistent.

While there are some significant advantages in considering APME and CPT-invariant thermodynamics, these advantages do not prove that it is the antisymmetric (and not symmetric) approach that corresponds to the real world. Absence of antimatter in the universe may have different explanations. Would it be possible to establish, at least in principle, which version of thermodynamics corresponds to the real world? It seems that the answer is generally positive but the following important points need to be taken into account.

The thermodynamic properties are not revealed in simple microscopic systems; this requires a system of sufficient size and complexity. A simple system placed into a thermodynamic environment does not create thermodynamic behaviour on its own, but is subject to the thermodynamic properties of the environment. Hence having relatively few isolated antistates or placing these antistates into a conventional thermodynamic environment does not create an antimatter-controlled thermodynamic system and does not solve the problem. The challenge is to create a thermodynamic object (i.e. object that is sufficiently complex and, conventionally, cannot be in a coherent state) that is made not from matter but from antimatter. This object should be sufficiently insulated from the environment so that the inter-object interactions are overwhelmingly stronger than any environmental interference. The difficulty of this task should not be underestimated but some encouraging news is arriving from high-energy colliders^20^,^21^. It is becoming possible to create antinuclei^22^ and even antiatoms^21^, but still only in very small quantities. The other possible object of interest is quark-gluon plasma, which can be created in high-energy collisions – despite its small size, this object seems to have some thermodynamic properties^20^,^22^. In any case, the progress in high-energy experiments moves forward quickly and a time when the thermodynamic properties of antimatter can be assessed experimentally may not be too far ahead.

## Conclusions

This work introduces the antisymmetric version of the Pauli master equation (APME). The symmetric version of the equation (SPME) is conventional. The proved H-theorem demonstrates consistency of the symmetric and antisymmetric versions of the PME with the symmetric and antisymmetric extensions of thermodynamics from matter into antimatter. The symmetric versions of these approaches are CP-invariant while the antisymmetric versions are CPT-invariant (under conditions specified in Proposition 1). These properties do not necessarily correspond to, and must not be confused with, microscopic invariant properties of the quantum Hamiltonians. The analysis of the present work demonstrates that SPME predicts evolution towards the same probabilities of matter and antimatter states, while APME points to full conversion of antimatter into matter. In the absence of experimental knowledge about thermodynamic properties of antimatter, we cannot make an experimentally justified choice in favour of the symmetric or antisymmetric versions, however continuing progress of high-energy physics will, hopefully, be able to resolve this dilemma in the future.

## Additional Information

**How to cite this article**: Klimenko, A. Y. Symmetric and antisymmetric forms of the Pauli master equation. *Sci. Rep.* **6**, 29942; doi: 10.1038/srep29942 (2016).
